# BCL6 inhibition: a promising approach to prevent germinal center-driven allo-immune responses

**DOI:** 10.3389/fimmu.2025.1667185

**Published:** 2025-10-31

**Authors:** Rens Kraaijeveld, Dennis A. Hesselink, Louisa Steines, Sebastiaan Heidt, Carla C. Baan

**Affiliations:** ^1^ Erasmus Medical Center (MC) Transplant Institute, Department of Internal Medicine, University Medical Center Rotterdam, Rotterdam, Netherlands; ^2^ Uniklinikum Erlangen - Department of Medicine 4 - Nephrology and Hypertension; Friedrich-Alexander-Universität (FAU) Erlangen-Nürnberg, Erlangen, Germany

**Keywords:** BCL6, transplantation, germinal center (GC), small molecule inhibitor, DLBCL

## Abstract

After solid organ transplantation, antibody-mediated rejection (AMR) is the most important cause of late allograft loss. Central in this process are donor-specific antibodies (DSAs) targeting mismatched Human Leukocyte Antigens (HLA) on recipient endothelial cells. Alloreactive B cells can directly bind to mismatched HLA molecules expressed by endothelial cells of a transplanted organ through their B cell receptor. Upon antigen recognition, B cells can differentiate into memory B cells and plasma cells producing class switched, high affinity DSAs. Cognate interaction between alloreactive follicular T helper cells (Tfh) and B cells, both expressing the transcription factor BCL6, is essential for long-lived plasma cell formation. Blockade of BCL6 by inhibitory compounds has emerged as a promising therapeutic strategy in the treatment of BCL6-expressing B cell lymphomas. Beyond its direct cytotoxic effects on malignant B cells, BCL6 inhibition also disrupts the function of germinal center B cells and impairs survival and activation of Tfh cells after immunization. These findings suggest that BCL6-targeting therapies may have potential as an immunosuppressive strategy in the context of organ transplantation, where controlling the humoral allo-immune response is essential to prevent graft rejection. This article reviews the mechanisms by which BCL6 controls Tfh and B cell differentiation and germinal center formation after organ transplantation. Finally, it outlines how newly discovered BCL6 inhibitory compounds might intervene with these B cell mediated immune responses.

## 1 Introduction

In solid organ transplantation, antibody-mediated rejection (AMR) is a major cause of late allograft loss. Antibodies directed against the donor, primarily targeting mismatched human leukocyte antigens (HLA), and in some cases, non-HLA antigens, play a key role in its pathophysiology. AMR is histologically characterized by microvascular inflammation (e.g., glomerulitis, peritubular capillaritis), along with the presence of circulating donor-specific antibodies (DSAs) (1, 2). These DSAs bind antigens expressed on endothelial cells of the allograft, triggering complement activation (often marked by C4d deposition) and antibody-dependent cellular cytotoxicity (ADCC). In addition, upon antibody binding, endothelial cells can become activated, resulting in further promotion of a pro-inflammatory milieu (3). Individuals with DSAs prior to (repeat) transplantation are at an increased risk for (hyper)acute rejection (4, 5). Therefore, organ donors to which HLA-specific antibodies are directed are usually excluded, but this significantly limits donor options (6). Despite strategies such as prioritization programs, HLA desensitization, and kidney exchange programs to mitigate risks and expand the donor pool, long waiting times for HLA compatible donor organs for these patients persist (7, 8). While DSAs are key mediators of AMR, their formation is rooted in the activation and differentiation of HLA-specific B cells, which lead to the formation of memory B cells and DSA-producing plasma cells. This activation is driven by antigen-specific T helper cells called follicular T helper cells (Tfh), which are specialized in providing B cell help. Thus, given their central joint role in AMR, B cells and Tfh cells represent promising targets for early treatment or prevention strategies of AMR. Given that there is an unmet need for immunosuppressive drugs that can treat established AMR and prevent DSA formation (9, 10), therapies that can target both cell types simultaneously are of great interest.

## 2 Relevant subsections

### 2.1 Current drugs used to regulate humoral immune responses in transplantation

With AMR being recognized as the main cause of graft loss in the long-term, interest in targeting the humoral arm of the alloimmune response has grown. Regardless, no approved therapies for prevention or treatment of AMR are currently available (11). The most commonly used combination of plasma exchange with intravenous immunoglobulins (IVIg) is being applied clinically in several variations (12). Despite this therapy being regarded as standard of care for acute, active AMR, the evidence for its efficacy is low (13). The B cell-depleting agent rituximab is often used in conjunction with plasma exchange and IVIg, but notwithstanding its frequent use, prospective trials have reported no benefit of the addition of rituximab to plasma exchange and IVIg (14, 15).

A recent addition to the armamentarium to target the humoral arm of the alloimmune response is the IgG-degrading enzyme of Streptococcus pyogenes (IdeS) (16). With this drug being highly efficient in cleaving circulating IgG within hours, it is conditionally approved by the European Medicines Agency (EMA) for kidney transplantation over a positive crossmatch (17). However, a recent study on the use of IdeS to treat AMR in kidney transplant recipients showed no clinical benefit over plasma exchange, despite superiority of IdeS to rapidly diminish DSA levels (18).

The proteasome inhibitor Bortezomib selectively targets plasma cells through apoptosis following the unfolded protein response, and is approved for the treatment of multiple myeloma. However, with only limited evidence for its efficacy in the transplantation setting and rather serious side effects (19, 20), the use of bortezomib for treatment of AMR has stalled. More recently, monoclonal antibodies targeting CD38 (daratumumab, felzartamab) have shown promising results in resolving AMR (21, 22), possibly due to targeting CD16^bright^ natural killer (NK) cells alongside plasma cells.

A role for the pleiotropic cytokine IL-6 in driving B cell activation and differentiation to antibody-producing plasma cells was suggested by murine studies on AMR (23). Nevertheless, while a small single-center clinical study on the treatment of chronic active AMR using the IL-6 targeting antibody tocilizumab showed good patient and graft survival (24), a recent phase 3 clinical trial using clazakizumab to treat chronic, active AMR was halted prematurely due the lack of efficacy. While IL-6 plays a role in early processes of Tfh differentiation by inducing BCL6 (25) and would potentially be targeted, directly targeting BCL6 may offer a more potent inhibition of both B cells and Tfh cells.

### 2.2 Role of BCL6 in germinal centers

Upon encountering allo-antigens, often in the form of donor HLA molecules, naïve B cells migrate to lymphoid follicles in secondary lymphoid organs. There, they interact with cognate alloreactive CD4^+^ Tfh cells that have been primed by antigen-presenting cells. This interaction, known as linked recognition, involves B cells presenting processed antigens as peptides to Tfh cells in HLA class II, and initiates Germinal Center (GC) formation. GCs are transient structures where B cells undergo somatic hypermutation (SHM) and class switch recombination (CSR), enhancing antibody affinity and function (26–28). Maintenance of both SHM and CSR requires signals provided by Tfh cells. Following priming by dendritic cells (DCs), prolonged allogeneic pressure induces expression of Inducible T-cell COStimulator (ICOS) in a subset of T helper cells. Subsequent ICOS–ICOSL interactions with DCs promote the upregulation of BCL6, CXCR5, and PD-1, driving Tfh cell differentiation and migration toward GCs by suppressing transcription factors associated with other helper T cell subsets (like TBET or GATA3) (29–33). Tfh cells are critical for B cell activation in GCs, driving differentiation and proliferation of GC B cells through the co-stimulatory signal CD40-CD154, coupled with IL-21 production, triggering CSR and SHM, respectively (34–36). Under the selective conditions of SHM, BCL6 in GC B cells enhances proliferative capacity and represses genes associated with DNA damage checkpoints, facilitating the DNA modifications required for CSR and SHM (31, 37). As GC B cells undergo CSR, increased BCR-antigen binding affinity by B cells and stronger CD40 signaling from Tfh cells drive downregulation of BCL6 in B cells, promoting differentiation into plasma cells. Thus, BCL6 is essential for Tfh cell identity and function within GCs, and maintaining effective SHM activity in GC B cells before their differentiation into effector plasma cells.

### 2.3 BCL6 structure and domains

**Figure 1:**
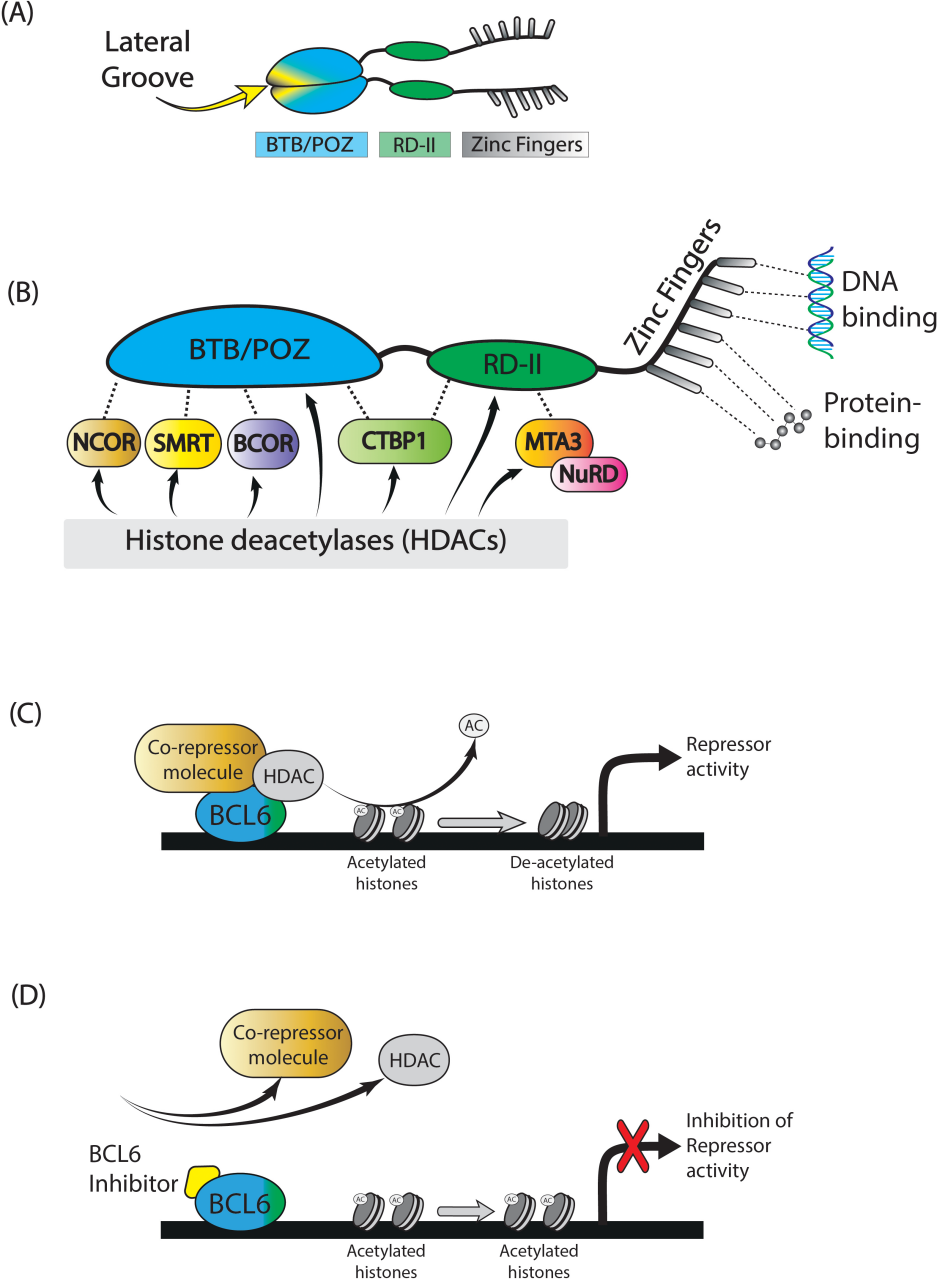
BCL6. BCL6 is a transcription factor which is actively functional in the form of a dimer. It consists of a BTB/POZ domain, RD-2 domain, and a zinc finger domain **(A)**. BCL6 functions by recruiting several co-repressor molecules, of which the most important are shown in **(B)**. In conjunction with histone deacetylase complex molecules, these co-repressor molecule complexes remove acetyl-groups of specific histones, resulting in repression of specific target genes **(C)**. The application of small molecule BCL6 inhibitors will block specific binding sites of the BCL6 BTB-domain, leaving it unable to form co-repressor molecules, thereby retaining target gene expression **(D)**. *Figure adapted from Crotty* et al (39).

BCL6 belongs to the BTB/POZ/zinc finger family of transcription factors (38) and consists of 3 domains, namely an N-terminal BTB repressor domain (Broad-complex, Tramtrack and Brick-a-brac), a second repressor (middle-) domain (RD2), and a C-terminal zinc finger domain (**Figure 1A**) (38). BCL6 functions in the form of a dimer, consisting of two BCL6 molecules coupled through their BTB domains. BCL6 binds directly to BCL6-specific DNA sequences through its zinc finger domain, enabling direct transcriptional repression (**Figure 1B**). This way, BCL6 competes with other transcription factors for DNA occupancy, and mediates protein-protein interactions. Secondly, BCL6 exerts repressive activity through its BTB and RD2 domains, by recruiting class 1 and 2 Histone deacetylase complexes (HDACs), and various co-repressor molecules, forming co-repressor complexes with the HDACs (**Figure 1B**). De-acetylation of histones targeted by these HDACS represses BCL6 target genes (e.g., BCL2, MYC, JUNB, FAIM3, HSP90AB, and IRF4), regulating cell differentiation (**Figure 1C**) (40, 41). The most significant co-repressor molecules associated with BCL6’s repressor domains are BCOR (BCL6 interacting corepressor) (42), NCOR1 (nuclear receptor corepressor), and SMRT1 (silencing mediator of retinoid and thyroid hormone receptors), all binding to the BTB domain; MTA3 (Metastasis associated 1 family, member 3), which binds to the RD2 domain; and CTBP1 (C-terminal-binding protein 1), binding to both BTB and RD2 domains (**Figure 1B**) (43). BTB domain driven co-repressor activity drives Tfh cell differentiation and B cell proliferation and survival, needed during SHM and CSR. Co-repressor activity by the RD2 domain plays a bigger role in early GC-commitment, through migration and pre-GC differentiation of B cells.

### 2.4 BCL6 inhibitory- or degrading-compounds

Several compounds have been developed to block BCL6 activity in the setting of B cell malignancies and autoimmune diseases, including peptides and small molecule BCL6 inhibitors. Both types of compounds block the BTB domain of BCL6, leading to inhibition of co-repressor function and subsequent increased expression of BCL6 target genes. Since the discovery of BCL6 as an important oncogene in B cell lymphoma formation, initial studies focused on treating B cell malignancies using BCL6 inhibition. Preclinical studies showed that BCL6 inhibition halted BCL6-positive tumor growth in mice, but also suppressed GC formation (44–46). Subsequent research showed that BCL6 inhibition is also able to reduce germinal center formation in the setting of autoimmunity and infection (47, 48). Also, this improved survival in sepsis, lowered HIV persistence by targeting infected Tfh cells, and halted lupus progression by reducing T and B cell activation. **Table 1** provides an overview of the most relevant advances in BCL6 inhibitor research.

**Table 1:** An overview of the most relevant developments in BCL6 inhibition research.

Novelty	Compound	Year	Study model	Main finding/advantage
Peptides	BBD peptide	2004	*In Vivo*, [mice]	Apoptosis of DLBCL cells, Abrogation of GCs
	RI-BPI	2009	*In Vivo*, [mice]	Kills human DLBCL cell-induced tumors
Small Molecules	79-6	2010	*In Vivo*, [mice]	First small molecule
	FX-1	2016	*In Vivo*, [mice]	Increased binding strength to BTB-domain compared to 79-6
	WK369	2023	*In Vivo*, [mice]	Stronger antitumor effects than FX-1
Prodrug of FX-1	AP-4-287	2021	*In Vivo*, [mice]	Increased solubility, represses Tfh and GC reactions
Orally available:	GSK137	2021	*In Vivo*, [mice]	Suppresses antibody responses
	WK500B	2022	*In Vivo*, [mice]	Inhibits GC formation and DLBCL cell growth
	OICR12694	2023	*In Vivo*, [mice/dogs]	Inhibits DLBCL cell growth, good oral bioavailability
Irreversible Binding:	TMX-2164	2020	*In Vitro*	Improved inhibitory activity and sustained target engagement, as compared to reversible inhibition
Degradation:	BI-3802	2017	*In Vitro*	BCL6 degradation is more potent than BCL6 inhibition
	‘PROTAC 15’	2018	*In Vitro*	BCL6 PROTAC shows incomplete BCL6 degradation
	DZ-837	2024	*In Vivo*, [mice]	Effective sustained BCL6 degradation as DLBCL treatment
Upcoming Clinical Trials:	ARV-393	2024	*In vivo*, [human]	A phase-1 study in adult participants with advanced non-Hodgkin’s lymphoma
	BMS-986458	2024	*In vivo*, [human]	A phase 1/2, multi-center study evaluating safety, tolerability, drug levels, and activity of BMS-986458 in lymphoma.

An overview of the more relevant research papers which have advanced the field on BCL6 inhibition and their corresponding information, categorized.

Inhibition of BCL6 activity can be established by several types of compounds, which bind to different parts of the BCL6 protein, mostly the BTB domain. Co-repressors such as SMRT1, BCOR, and NCOR1 exert their activity by binding to the lateral groove of the BTB domain of two BCL6 molecules as a dimer (**Figure 1A**). Inhibitors designed to mimic co-repressor binding disrupt BCL6’s activity to form co-repressor complexes, blocking BCL6 activity (**Figure 1D**). Additionally, BCL6 peptide inhibitors like BTB binding domain motif (BBD) peptides, and retro-inverted BCL6 peptide inhibitor (RI-BPI) peptides can block co-repressor activity and have been shown in multiple studies to inhibit the growth of B cell lymphoma cell lines *in vitro* and resulting tumor growth in mouse models (46, 49). However, due to their large size and charged nature, their ability to pass through cell membranes is limited. Therefore, smaller molecules with lower molecular weights have been explored as potential BCL6 inhibitors.

Through computer-aided drug design, the small molecule inhibitor 79–6 was developed to disrupt the interaction of BCL6 with NCOR and SMRT co-repressor complexes, restoring BCL6 target gene expression. This inhibitor was shown to specifically kill BCL6-positive diffuse large B cell Lymphoma (DLBCL) cell lines (50). However, due to 79-6’s relatively low binding affinity for the BTB domain compared to endogenous co-repressors, the more potent inhibitor FX-1 was developed, a molecule with increased binding affinity for the BTB domain (44). Small molecules like 79–6 and FX-1 disrupt interaction of BCL6 with NCOR/SMRT, restoring BCL6 target gene expression. Additionally, 79–6 and FX-1 were both shown to selectively kill BCL6-positive DLBCL cell lines (44, 50). These compounds have undergone testing to assess their toxicity *in vivo*, and showed no evidence of harmful effects in animal models (44, 46, 50). More recently, the small molecules WK369 and WK692 have been developed, both suppressing the transcriptional inhibitory activity of BCL6 *in vitro* and *in vivo (* 51, 52). In a mouse model of ovarian cancer, WK369 prevented cancer growth and suppressed BCL6-driven AKT and MEK/ERK signaling, which are intracellular pathways linked to cancer progression. WK692 was shown to inhibit DLBCL growth *in vitro* and abrogated GC formation *in vivo.* WK692 also induced stronger re-expression of BCL6 target genes, as compared to FX-1 (51, 52).

Concomitantly, existing small molecule inhibitors were optimized to improve key pharmacological aspects. BTB-specific inhibitors suffer from poor water solubility. To improve solubility, AP-4–287 was developed as a prodrug of FX-1, increasing aqueous solubility 150-fold. Although AP-4–287 retained the ability to inhibit Tfh cell differentiation and GC formation, the pro-drug required higher concentrations to achieve the same effect as FX-1, and showed a shorter half-life (53). Additionally, orally available small molecule compounds have been developed (GSK137, WK500b, OICR12694), that still effectively inhibit the growth of DLBCL cell lines in mouse models (45, 54, 55).

To enhance the suppression of BCL6, target protein degradation and irreversible binding have been explored. At first, Kerres et al. simultaneously developed two agents, BI-3812 and BI-3802. While BI-3812 functions as a reversible BCL6 BTB domain inhibitor, BI-3802 unexpectedly induced BCL6 degradation (56). This occurs through polymerization of BCL6, leading to degradation through the ubiquitin-proteasome system (UPS). Proteasomal degradation leads to profound re-expression of BCL6 target genes and anti-proliferative effects comparable to genetic knockout of BCL6 in DLBCL models (57). Later, to harness UPS-mediated degradation, a BCL6-targeting PROTAC (Proteolysis Targeting Chimera) was developed, which is a small molecule that tags unwanted proteins (in this case BCL6) for degradation. Unfortunately, this compound only resulted in partial BCL6 depletion, rendering it no more effective than standard inhibitors (58). In contrast, TMX-2164, a covalent inhibitor, irreversibly binds to amino acid Tyr58 in the lateral groove, offering greater potency than BI-3812 without resulting in active degradation (59). Lastly, researchers developed a novel BCL6-targeting PROTAC (DZ-837) featuring an N-phenyl-4-pyrimidinamine scaffold, recruiting UPS in a direct manner for targeted BCL6 degradation (60). This approach was shown to effectively eliminate BCL6 in DLBCL cells, leading to sustained re-expression of downstream genes, while also inducing G1 phase arrest, ultimately suppressing tumor growth. With DZ-837 and other emerging PROTACs, targeted BCL6 degradation is evolving into a powerful therapeutic strategy, further validating targeted protein degradation as a therapeutic approach for targeting BCL6.

Finally, two novel agents, BMS-986458, a BCL6 degrading compound, and ARV-393, a BCL6 targeting PROTAC, are currently under clinical investigation to assess their safety and efficacy in targeting BCL6 in relapsed/refractory non-hodgkin lymphoma patients (61, 62). These clinical trials represent significant advances in the development of targeted therapies against BCL6.

### 2.5 Application in transplantation

Since BCL6 is a key regulator of malignant B cell growth, most studies on pharmacologically targeting BCL6 are aimed at eliminating BCL6-expressing lymphomas *in vivo (* 44–46, 50). For transplant patients, there is a risk of developing posttransplant lymphoproliferative disorder(PTLD). Some subtypes of these tumors (e.g. PT-DLBCL) express BCL6. In these cases, BCL6 inhibition might prove useful in a comparable way to treatment of DLBCL (63). Interestingly, BCL6 inhibition was also found to inhibit BCL6-driven immune responses, like T cell-dependent B cell activation in GCs (52, 53). Therefore, the scope of BCL6 inhibition research has expanded to suppressing immune responses driven by BCL6-expressing B cells and Tfh cells, including its application in infectious diseases and autoimmunity. In these studies, application of BCL6 inhibition was shown to exert anti-inflammatory effects in LPS-driven sepsis, reduce HIV infected CD4^+^ T cell numbers, and to suppress Tfh cell activation and GC formation (47, 48, 53, 64).

Because of the central role of BCL6 in GC responses, BCL6-inhibition has also been studied in allo-immune responses (65). In an *in vitro* model of allo-antigen stimulated Tfh and B cells, early addition (day 0) of the small molecule inhibitor 79–6 resulted in inhibition of both alloantigen-driven B cell activation and plasmablast formation, while late addition (day 3 or later) did not (66), highlighting the importance of targeting BCL6 at early stages of cell differentiation. This aligns well with the findings of Cai et al., who showed that following stimulation with red blood cells *in vivo* (RBCs), pre-established antibody responses were not inhibited by BCL6 blockade, likely because plasma cells were already present prior to treatment (53).

Paz et al. studied the effect of BCL6 inhibition in two mouse models of chronic GvHD (cGvHD) with different pathologies (67). In a GC independent model of sclerodermatous cGvHD, BCL6 inhibition by 79–6 was unable to inhibit the predominant Th1 and Th17 responses, showing the GC restricted activity of BCL6. Additionally, in a GC-driven Bronchiolitis Obliterans cGvHD model, 79–6 did not affect splenic Tfh cell numbers, but did reduce splenic GC B cell numbers and IgG deposition in the lungs, thereby preventing pulmonary dysfunction (67). These data stress that BCL6 inhibition is strictly limited to GC-driven responses, and may thus not affect extrafollicular plasmablast activity (67, 68).

Alternatively, Chen et al. set up a sclerodermatous cGvHD model that does rely on GC activity. Here, BCL6 inhibition by 79–6 alleviated cGvHD symptoms, prolonged survival, and reduced fibrosis in lungs and neck skin (69). Additionally, 79–6 treatment significantly decreased peripheral and splenic Tfh cell and GC B cell numbers and was associated with a reduction of in IgG deposition in the spleens (69). Although both studies employed sclerodermatous cGVHD models, Chen et al. infused total spleen cells—including BCL6-expressing B cells—while Paz et al. used only splenic T cells. This would account for the stronger GC responses observed in Chen’s model. In short, both studies suggest a key role for BCL6-expressing cells in the development of GC driven cGVHD and highlight the significance of BCL6 inhibition in the prevention of such allo-immune responses. More recently, the potential of BCL6 inhibition to prevent heart allograft rejection was investigated. Xia et al. established an allogeneic heart transplantation mouse model, in which BCL6 inhibition was established by 25 mg/kg of FX-1 on the first three days after transplantation. While formation of Tfh cells was inhibited by FX-1, no increase in graft survival, nor any changes in graft pathology were detected. In contrast, a model was set up where administration of CTLA-4-Ig on the first day after transplant prolonged graft survival and delayed Tfh formation to around 28 days (70). When FX-1 was infused in this model, graft survival was significantly increased during this time period, with reduced vascular occlusions and reduced fibrotic areas in the graft. Additionally, GC reactions were inhibited at this time point, as shown by a reduction in Tfh cell and GC B cell numbers in the spleen. And as a result, splenic plasma cell numbers and DSA levels (MFI) were significantly reduced (70).

### 2.6 Future directions, conclusions and remarks

Preclinical studies have shown that blocking the BTB groove of BCL6 with peptides or small molecules reduces corepressor recruitment and transcription of BCL6, resulting in increased expression of BCL6 target genes and reduced cell growth (44, 46, 50, 71). Promising results have been reported from murine models, with these compounds killing BCL6-positive DLBCL derived tumors and decreasing GC formation. Beyond B cell lymphomas, BCL6 inhibition also impacts Tfh cells and GC B cells and may regulate immune responses during infectious disease (48, 64), auto-immunity (47), or allo-immune responses (67, 69, 70). In the field of solid organ transplantation, the inhibition of BCL6 presents a potential new way for the prevention of AMR. As BCL6-inhibition plays a crucial role in regulating Tfh cells as well as B cells, targeting BCL6 could help reduce the incidence of chronic rejection in organ transplant patients.

A critical note is that the timing of BCL6 inhibition may be of the essence. Whereas BCL6 plays a pivotal role in differentiation of Tfh and GC B cells and GC formation, BCL6-expression becomes less relevant once differentiation progresses, limiting the therapeutic window for intervention. Based on this, we expect BCL6 inhibition to be best used as a method to prevent B cell activation instead of treatment of an ongoing immune response. Firstly, BCL6 inhibition might be used as induction therapy in the case of sensitized patients, as dormant HLA-specific memory B cells are likely to be present (72). However, since in this case established DSAs and DSA producing plasma cells are not removed, targeting DSAs (e.g. plasmapheresis, Imlifidase) and/or plasma cell depletion therapy should be considered. Secondly, BCL6 inhibition could possibly be used as maintenance immunosuppression, in which it could prevent DSA formation. It should be noted however, that in combination with T cell immunosuppression, the patient is exposed to prolonged elevated risk to infections and lowered vaccine responses. To investigate this matter further, future studies should determine the optimal timing of BCL6 inhibition in a transplant setting and for what clinical indication such intervention could be used (prevention *vs*. treatment of established AMR). Given its dual role in regulating Tfh and B cell responses, BCL6 inhibition could pave the way for more targeted and effective immunomodulatory therapies in transplantation. While clinical studies in humans are ongoing in the field of oncology, further research is needed to establish efficacy and safety in transplantation. Nonetheless, promising preclinical data suggest that BCL6 inhibition could become a valuable strategy for reducing AMR and improving long-term graft survival.

## References

[1] Montgomery RA Loupy A Segev DL. Antibody-mediated rejection: New approaches in prevention and management. Am J Transplant. (2018) 18:3–17. doi: 10.1111/ajt.14584, PMID: 29292861

[2] Einecke G Sis B Reeve J Mengel M Campbell PM Hidalgo LG. Antibody-mediated microcirculation injury is the major cause of late kidney transplant failure. Am J Transplant. (2009) 9:2520–31. doi: 10.1111/j.1600-6143.2009.02799.x, PMID: 19843030

[3] Cross AR Glotz D Mooney N. The role of the endothelium during Antibody-mediated rejection: From victim to accomplice. Front Immunol. (2018) 9:1–7. doi: 10.3389/fimmu.2018.00106, PMID: 29434607 PMC5796908

[4] Betjes MGH Sablik KS Otten HG Roelen DL Claas FH de Weerd A. Pretransplant donor-specific anti-HLA antibodies and the risk for rejection-related graft failure of kidney allografts. J Transplant. (2020) 2020:1–10. doi: 10.1155/2020/5694670, PMID: 32099669 PMC7008278

[5] Wehmeier C Amico P Sidler D Wirthmüller U Hadaya K Ferrari-Lacraz S. Pre-transplant donor-specific HLA antibodies and risk for poor first-year renal transplant outcomes: results from the Swiss Transplant Cohort Study. Transpl Int. (2021) 34:2755–68. doi: 10.1111/tri.14119, PMID: 34561920

[6] de Ferrante H Smeulders B Tieken I Heidt S Haasnoot GW Claas FHJ. Immunized patients face reduced access to transplantation in the eurotransplant kidney allocation system. Transplantation. (2023) 107:2247–54. doi: 10.1097/TP.0000000000004687, PMID: 37291726

[7] Choi AY Manook M Olaso D Ezekian B Park J Freischlag K. Emerging new approaches in desensitization: targeted therapies for HLA sensitization. Front Immunol. (2021) 12:694763. doi: 10.3389/fimmu.2021.694763, PMID: 34177960 PMC8226120

[8] Kanaan N Mourad M Goffin E. Living donor exchange program in kidney transplantation: an underexploited organ resource. Transplantation. (2024) 108:2294–5. doi: 10.1097/TP.0000000000005202, PMID: 39279048

[9] Jordan SC Vo A Tyan D Toyota M. Desensitization therapy with intravenous gammaglobulin (IVIG): applications in solid organ transplantation. Trans Am Clin Climatol Assoc. (2006) 117:199–211; discussion 211., PMID: 18528474 PMC1500934

[10] Everly JJ Walsh RC Alloway RR Woodle ES. Proteasome inhibition for antibody-mediated rejection. Curr Opin Organ Transplant. (2009) 14:662–6. doi: 10.1097/MOT.0b013e328330f304, PMID: 19667989

[11] Schinstock CA Mannon RB Budde K Chong AS Haas M Knechtle S. Recommended treatment for antibody-mediated rejection after kidney transplantation: the 2019 expert consensus from the transplantion society working group. Transplantation. (2020) 104:911–22. doi: 10.1097/TP.0000000000003095, PMID: 31895348 PMC7176344

[12] Wan SS Ying TD Wyburn K Roberts DM Wyld M Chadban SJ. The treatment of antibody-mediated rejection in kidney transplantation. Transplantation. (2018) 102:557–68. doi: 10.1097/TP.0000000000002049, PMID: 29315141

[13] Eckardt KU Kasiske BL Zeier MG. Special issue: KDIGO clinical practice guideline for the care of kidney transplant recipients. Am J Transplant. (2009) 9:S1–155. doi: 10.1111/j.1600-6143.2009.02834.x, PMID: 19845597

[14] Sautenet B Blancho G Büchler M Morelon E Toupance O Barrou B. One-year results of the effects of rituximab on acute antibody-mediated rejection in renal transplantation. Transplantation. (2016) 100:391–9. doi: 10.1097/TP.0000000000000958, PMID: 26555944

[15] Moreso F Crespo M Ruiz JC Torres A Gutierrez-Dalmau A Osuna A. Treatment of chronic antibody mediated rejection with intravenous immunoglobulins and rituximab: A multicenter, prospective, randomized, double-blind clinical trial. Am J Transplant. (2018) 18:927–35. doi: 10.1111/ajt.14520, PMID: 28949089

[16] Lonze BE Tatapudi VS Weldon EP Min ES Ali NM Deterville CL. IdeS (Imlifidase): A novel agent that cleaves human igG and permits successful kidney transplantation across high-strength donor-specific antibody. Ann Surg. (2018) 268:488–96. doi: 10.1097/SLA.0000000000002924, PMID: 30004918

[17] Jordan SC Maldonado AQ Lonze BE Sjöholm K Lagergren A Montgomery RA. Long-term outcomes at 5 years posttransplant in imlifidase-desensitized kidney transplant patients. Am J Transplant. (2025) 25:878–80. doi: 10.1016/j.ajt.2024.11.029, PMID: 39643005

[18] Halleck F Böhmig GA Couzi L Rostaing L Einecke G Lefaucheur C. A randomized trial comparing imlifidase to plasmapheresis in kidney transplant recipients with antibody-mediated rejection. Clin Transplant. (2024) 38. doi: 10.1111/ctr.15383, PMID: 39023092

[19] Eskandary F Regele H Baumann L Bond G Kozakowski N Wahrmann M. A randomized trial of bortezomib in late antibody-mediated kidney transplant rejection. J Am Soc Nephrol. (2018) 29:591–605. doi: 10.1681/ASN.2017070818, PMID: 29242250 PMC5791086

[20] Moreno Gonzales MA Gandhi MJ Schinstock CA Moore NA Smith BH Braaten NY. 32 doses of bortezomib for desensitization is not well tolerated and is associated with only modest reductions in anti-HLA antibody. Transplantation. (2017) 101:1222–7. doi: 10.1097/TP.0000000000001330, PMID: 27379560 PMC4935916

[21] Doberer K Kläger J Gualdoni GA Mayer KA Eskandary F Farkash EA. CD38 antibody daratumumab for the treatment of chronic active antibody-mediated kidney allograft rejection. Transplantation. (2021) 105:451–7. doi: 10.1097/TP.0000000000003247, PMID: 32235256

[22] Mayer KA Schrezenmeier E Diebold M Halloran PF Schatzl M Schranz S. A randomized phase 2 trial of felzartamab in antibody-mediated rejection. N Engl J Med. (2024) 391:122–32. doi: 10.1056/NEJMoa2400763, PMID: 38804514

[23] Wu G Chai N Kim I Klein AS Jordan SC. Monoclonal anti-interleukin-6 receptor antibody attenuates donor-specific antibody responses in a mouse model of allosensitization. Transpl Immunol. (2013) 28:138–43. doi: 10.1016/j.trim.2013.03.003, PMID: 23562586

[24] Choi J Aubert O Vo A Loupy A Haas M Puliyanda D. Assessment of tocilizumab (Anti–interleukin-6 receptor monoclonal) as a potential treatment for chronic antibody-mediated rejection and transplant glomerulopathy in HLA-sensitized renal allograft recipients. Am J Transplant. (2017) 17:2381–9. doi: 10.1111/ajt.14228, PMID: 28199785

[25] Choi YS Eto D Yang JA Lao C Crotty S. Cutting edge: STAT1 is required for IL-6–mediated bcl6 induction for early follicular helper cell differentiation. J Immunol. (2013) 190:3049–53. doi: 10.4049/jimmunol.1203032, PMID: 23447690 PMC3626564

[26] Kwun J Manook M Page E Burghuber C Hong J Knechtle SJ. Crosstalk between T and B cells in the germinal center after transplantation. Transplantation. (2017) 101:704–12. doi: 10.1097/TP.0000000000001588, PMID: 27906827 PMC5360505

[27] Nutt SL Tarlinton DM. Germinal center B and follicular helper T cells: siblings, cousins or just good friends? Nat Immunol. (2011) 12:472–7. doi: 10.1038/ni.2019, PMID: 21739669

[28] de Graav GN Dieterich M Hesselink DA Boer K Clahsen-van Groningen MC Kraaijeveld R. Follicular T helper cells and humoral reactivity in kidney transplant patients. Clin Exp Immunol. (2015) 180:329–40. doi: 10.1111/cei.12576, PMID: 25557528 PMC4408167

[29] Choi YS Kageyama R Eto D Escobar TC Johnston RJ Monticelli L. ICOS Receptor Instructs T Follicular Helper Cell versus Effector Cell Differentiation via Induction of the Transcriptional Repressor Bcl6. Immunity. (2011) 34:932–46. doi: 10.1016/j.immuni.2011.03.023, PMID: 21636296 PMC3124577

[30] Kitano M Moriyama S Ando Y Hikida M Mori Y Kurosaki T. Bcl6 protein expression shapes pre-germinal center B cell dynamics and follicular helper T cell heterogeneity. Immunity. (2011) 34:961–72. doi: 10.1016/j.immuni.2011.03.025, PMID: 21636294

[31] Nurieva RI Chung Y Martinez GJ Yang XO Tanaka S Matskevitch TD. Bcl6 mediates the development of T follicular helper cells. Science. (2009) 325:1001–5. doi: 10.1126/science.1176676, PMID: 19628815 PMC2857334

[32] Hatzi K Nance JP Kroenke MA Bothwell M Haddad EK Melnick A. BCL6 orchestrates Tfh cell differentiation via multiple distinct mechanisms. J Exp Med. (2015) 212:539–53. doi: 10.1084/jem.20141380, PMID: 25824819 PMC4387288

[33] Crotty S Johnston RJ Schoenberger SP. Effectors and memories: Bcl-6 and Blimp-1 in T and B lymphocyte differentiation. Nat Immunol. (2010) 11:114–20. doi: 10.1038/ni.1837, PMID: 20084069 PMC2864556

[34] De Silva NS Klein U. Dynamics of B cells in germinal centres. Nat Rev Immunol. (2015) 15:137–48. doi: 10.1038/nri3804, PMID: 25656706 PMC4399774

[35] Kroenke MA Eto D Locci M Cho M Davidson T Haddad E. Bcl6 and Maf cooperate to instruct human follicular helper CD4 T cell (Tfh) differentiation. Bone. (2008) 23:1–7. doi: 10.1038/jid.2014.371, PMID: 22427637 PMC3324673

[36] Basso K Dalla-Favera R. Roles of BCL6 in normal and transformed germinal center B cells. Immunol Rev. (2012) 247:172–83. doi: 10.1111/j.1600-065X.2012.01112.x, PMID: 22500840

[37] Ranuncolo SM Polo JM Dierov J Singer M Kuo T Greally J. Bcl-6 mediates the germinal center B cell phenotype and lymphomagenesis through transcriptional repression of the DNA-damage sensor ATR. Nat Immunol. (2007) 8:705–14. doi: 10.1038/ni1478, PMID: 17558410

[38] Huret JL Ahmad M Arsaban M Bernheim A Cigna J Desangles F. Atlas of genetics and cytogenetics in oncology and haematology in 2013. Nucleic Acids Res. (2013) 41:D920–4. doi: 10.1093/nar/gks1082, PMID: 23161685 PMC3531131

[39] Crotty S. T follicular helper cell differentiation, function, and roles in disease. Immunity. (2014) 41:529–42. doi: 10.1016/j.immuni.2014.10.004, PMID: 25367570 PMC4223692

[40] Shaffer AL Yu X He Y Boldrick J Chan EP Staudt LM. BCL-6 represses genes that function in lymphocyte differentiation, inflammation, and cell cycle control. Immunity. (2000) 13:199–212. doi: 10.1016/S1074-7613(00)00020-0, PMID: 10981963

[41] Hatzi K Jiang Y Huang C Garrett-bakelman F Micah D Giannopoulou EG. A hybrid mechanism of action for BCL6 in B cells defined by formation of functionally distinct complexes at enhancers and promoters. Cell reports (2014) 4:578–88. doi: 10.1016/j.celrep.2013.06.016, PMID: 23911289 PMC3854650

[42] Huynh KD Fischle W Verdin E Bardwell VJ. BCoR, a novel corepressor involved in BCL-6 repression. Genes Dev. (2000) 14:1810–23. doi: 10.1101/gad.14.14.1810, PMID: 10898795 PMC316791

[43] Huang CX Melnick A. Mechanisms of action of BCL6 during germinal center B cell development. Sci China Life Sci. (2015) 58:1226–32. doi: 10.1007/s11427-015-4919-z, PMID: 26566802

[44] Cardenas MG Yu W Beguelin W Teater MR Geng H Goldstein RL. Rationally designed BCL6 inhibitors target activated B cell diffuse large B cell lymphoma. J Clin Invest. (2016) 126:3351–62. doi: 10.1172/JCI85795, PMID: 27482887 PMC5004937

[45] Xing Y Guo W Wu M Xie J Huang D Hu P. An orally available small molecule BCL6 inhibitor effectively suppresses diffuse large B cell lymphoma cells growth *in vitro* and in *vivo* . Cancer Lett. (2022) 529:100–11. doi: 10.1016/j.canlet.2021.12.035, PMID: 34990752

[46] Cerchietti LC Yang SN Shaknovich R Hatzi K Polo JM Chadburn A. A peptomimetic inhibitor of BCL6 with potent antilymphoma effects *in vitro* and *in vivo* . Blood. (2009) 113:3397–405. doi: 10.1182/blood-2008-07-168773, PMID: 18927431 PMC2668844

[47] Venkatadri R Sabapathy V Dogan M Mohammad S Harvey SE Simpson SR. Targeting Bcl6 in the TREX1 D18N murine model ameliorates autoimmunity by modulating T-follicular helper cells and germinal center B cells. Eur J Immunol. (2022) 52:825–34. doi: 10.1002/eji.202149324, PMID: 35112355 PMC9089306

[48] Cai Y Abdel-Mohsen M Tomescu C Xue F Wu G Howell BJ. BCL6 inhibitor-mediated downregulation of phosphorylated SAMHD1 and T cell activation are associated with decreased HIV infection and reactivation. J Virol. (2019) 93:1–15. doi: 10.1128/JVI.01073-18, PMID: 30355686 PMC6321929

[49] Ghetu AF Corcoran CM Cerchietti L Bardwell VJ Melnick A Privé GG. Structure of a BCOR corepressor peptide in complex with the BCL6 BTB domain dimer. Mol Cell. (2008) 29:384–91. doi: 10.1016/j.molcel.2007.12.026, PMID: 18280243 PMC2665293

[50] Cerchietti LC Ghetu AF Zhu X Da Silva GF Zhong S Matthews M. A small-molecule inhibitor of BCL6 kills DLBCL cells *in vitro* and in *vivo* . Cancer Cell. (2010) 17:400–11. doi: 10.1016/j.ccr.2009.12.050, PMID: 20385364 PMC2858395

[51] Wu M Xie J Xing Y Zhang L Chen H Tang B. Selectively targeting BCL6 using a small molecule inhibitor is a potential therapeutic strategy for ovarian cancer. Int J Biol Sci. (2024) 20:486–501. doi: 10.7150/ijbs.86303, PMID: 38169532 PMC10758095

[52] Xing Y Guo W Wu M Xie J Huang D Hu P. A small-molecule BCL6 inhibitor as an anti-proliferative agent for diffuse large B-cell lymphoma. Mol Cancer Ther. (2025) 24:81–92. doi: 10.1158/1535-7163.MCT-23-0830, PMID: 39387112

[53] Cai Y Poli ANR Vadrevu S Gyampoh K Hart C Ross B. BCL6 BTB-specific inhibitor reversely represses T-cell activation, Tfh cells differentiation, and germinal center reaction in *vivo* . Eur J Immunol. (2021) 51:2441–51. doi: 10.1002/eji.202049150, PMID: 34287839 PMC8745002

[54] Pearce AC Bamford MJ Barber R Bridges A Convery MA Demetriou C. GSK137, a potent small-molecule BCL6 inhibitor with *in vivo* activity, suppresses antibody responses in mice. J Biol Chem. (2021) 297:100928. doi: 10.1016/j.jbc.2021.100928, PMID: 34274316 PMC8350397

[55] Mamai A Chau AM Wilson BJ Watson ID Joseph BB Subramanian PR. Discovery of OICR12694: A novel, potent, selective, and orally bioavailable BCL6 BTB inhibitor. ACS Med Chem Lett. (2023) 14:199–210. doi: 10.1021/acsmedchemlett.2c00502, PMID: 36793435 PMC9923840

[56] Kerres N Steurer S Schlager S Bader G Berger H Caligiuri M. Chemically induced degradation of the oncogenic transcription factor BCL6. Cell Rep. (2017) 20:2860–75. doi: 10.1016/j.celrep.2017.08.081, PMID: 28930682

[57] Słabicki M Yoon H Koeppel J Nitsch L Roy Burman SS Di Genua C. Small-molecule-induced polymerization triggers degradation of BCL6. Nature. (2020) 588:164–8. doi: 10.1038/s41586-020-2925-1, PMID: 33208943 PMC7816212

[58] McCoull W Cheung T Anderson E Barton P Burgess J Byth K. Development of a novel B-cell lymphoma 6 (BCL6) PROTAC to provide insight into small molecule targeting of BCL6. ACS Chem Biol. (2018) 13:3131–41. doi: 10.1021/acschembio.8b00698, PMID: 30335946

[59] Teng M Ficarro SB Yoon H Che J Zhou J Fischer ES. Rationally designed covalent BCL6 inhibitor that targets a tyrosine residue in the homodimer interface. ACS Med Chem Lett. (2020) 11:1269–73. doi: 10.1021/acsmedchemlett.0c00111, PMID: 32551010 PMC7294706

[60] Mi D Li C Li Y Yao M Li Y Hong K. Discovery of novel BCL6-Targeting PROTACs with effective antitumor activities against DLBCL *in vitro* and in *vivo* . Eur J Med Chem. (2024) 277:116789. doi: 10.1016/j.ejmech.2024.116789, PMID: 39208743

[61] Squibb Bristol Myers. A phase 1/2, multi-center, open-label, dose-finding study to evaluate the safety, tolerability, pharmacokinetics, pharmacodynamics, and preliminary efficacy of BMS-986458, alone and in combination with anti-lymphoma agents in participants with relapsed/re. clinicaltrials.gov (2023). Available online at: https://clinicaltrials.gov/study/NCT06090539.

[62] Arvinas inc. A phase 1 first in human study of ARV-393 in adult participants with advanced non-hodgkin’s lymphoma. clinicaltrials.gov (2024). Available online at: https://clinicaltrials.gov/study/NCT06393738.

[63] Baram DV Asaulenko ZP Spiridonov IN Krivolapov YA. WHO classification of tumors of hematopoietic and lymphoid tissues, 2022 (5th edition): lymphoid tumors. Russ J Arch Pathol. (2023) 85:24. doi: 10.17116/patol20238504124, PMID: 37530187

[64] Zhang H Qi X Wu J Huang X Zhang A Chen S. BCL6 inhibitor FX1 attenuates inflammatory responses in murine sepsis through strengthening BCL6 binding affinity to downstream target gene promoters. Int Immunopharmacol. (2019) 75:105789. doi: 10.1016/j.intimp.2019.105789, PMID: 31401377

[65] Boiko JR Hill GR. Chronic graft-versus-host disease: immune insights, therapeutic advances, and parallels for solid organ transplantation. Transplantation. (2024) 00:1–12. doi: 10.1097/TP.0000000000005298, PMID: 39682018 PMC12097962

[66] Kraaijeveld R Hesselink DA Dieterich M van den Bosch TPP Heidt S Baan CC. Small molecule BCL6-inhibition suppresses follicular T helper cell differentiation and plasma blast formation. Hum Immunol. (2025) 86:111242. doi: 10.1016/j.humimm.2025.111242, PMID: 39903994

[67] Paz K Flynn R Du J Qi J Luznik L Maillard I. Small-molecule BCL6 inhibitor effectively treats mice with nonsclerodermatous chronic graft-versus-host disease. Blood. (2019) 133:94–9. doi: 10.1182/blood-2018-03-839993, PMID: 30279226 PMC6318432

[68] Su H Zhang CY Lin Jh Hammes HP Zhang C. The role of long-lived plasma cells in antibody-mediated rejection of kidney transplantation: an update. Kidney Dis. (2019) 5:211–9. doi: 10.1159/000501460, PMID: 31768378 PMC6873015

[69] Chen X Wang Y Huang X Geng S Li C Zeng L. Targeting Bcl-6 prevents sclerodermatous chronic graft-versus-host disease by abrogating T follicular helper differentiation in mice. Int Immunopharmacol. (2023) 117:109746. doi: 10.1016/j.intimp.2023.109746, PMID: 36827923

[70] Xia Y Jin S Wu Y. Small-molecule BCL6 inhibitor protects chronic cardiac transplant rejection and inhibits T follicular helper cell expansion and humoral response. Front Pharmacol. (2023) 14:1140703/full. doi: 10.3389/fphar.2023.1140703/full, PMID: 37007047 PMC10063191

[71] Polo JM Dell’Oso T Ranuncolo SM Cerchietti L Beck D Da Silva GF. Specific peptide interference reveals BCL6 transcriptional and oncogenic mechanisms in B-cell lymphoma cells. Nat Med. (2004) 10:1329–35. doi: 10.1038/nm1134, PMID: 15531890

[72] Wehmeier C Karahan GE Krop J de Vaal Y Langerak-Langerak J Binet I. Donor-specific B cell memory in alloimmunized kidney transplant recipients: first clinical application of a novel method. Transplantation. (2020) 104:1026–32. doi: 10.1097/TP.0000000000002909, PMID: 31397804

