# Supplementary material for: BCL6 inhibition: a promising approach to prevent germinal center-driven allo-immune responses
Source: Front Immunol. 2025 Oct 31;16:1667185. doi: 10.3389/fimmu.2025.1667185 (PMC12615185; doi:10.3389/fimmu.2025.1667185)
Supplement: Supplementary file 1 [file Image1.pdf]

Supplementary Material

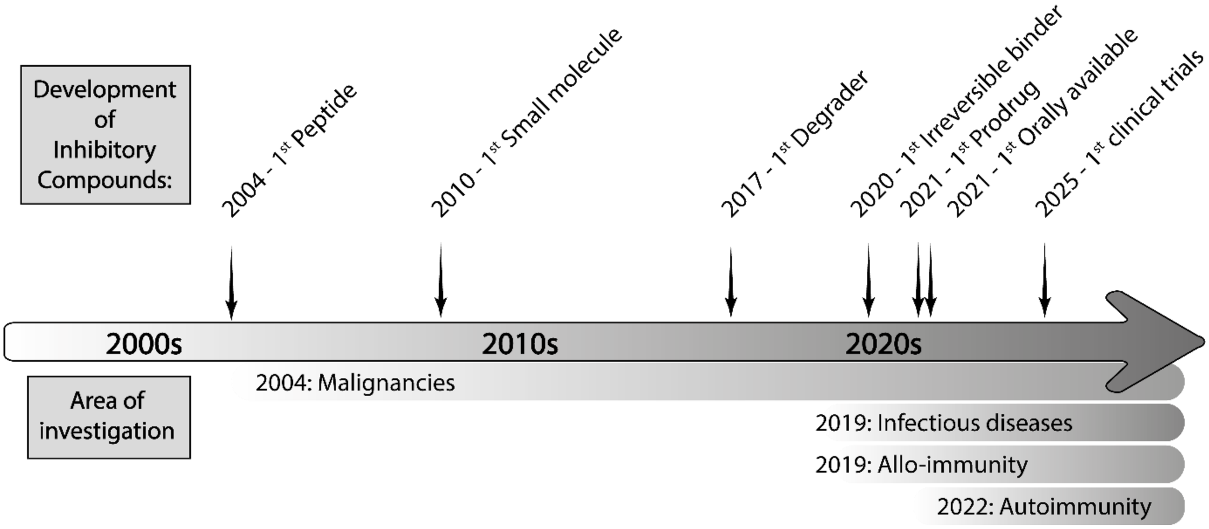

Supplementary Figure 1: Advancements in BCL6 inhibitory research
A timeline showing developments in the field of BCL6 inhibition research. The upper part of the timeline shows the time points at which new inhibitory compounds were developed. The lower part of the timeline highlights key milestones where BCL6 inhibitory compounds were first explored as therapeutic applications for different diseases.
